# One-year follow-up of accelerated transepithelial corneal collagen cross-linking for progressive pediatric keratoconus

**DOI:** 10.1186/s12886-018-0739-9

**Published:** 2018-03-09

**Authors:** Mi Tian, Weijun Jian, Ling Sun, Yang Shen, Xiaoyu Zhang, Xingtao Zhou

**Affiliations:** Department of Ophthalmology, Eye and ENT Hospital of Fudan University, Myopia Key Laboratory of the Health Ministry, No.19 Baoqing Road, Shanghai, 200031 People’s Republic of China

**Keywords:** Accelerated transepithelial corneal collagen cross-linking, Keratoconus, Pediatrics

## Abstract

**Background:**

Keratoconus typically presents in the teenage years and is more advanced in younger patients when compared with adults. In the present study, we aimed to assess the safety and efficacy of accelerated transepithelial corneal collagen cross-linking (ATE-CXL) in children with progressive keratoconus.

**Methods:**

In this retrospective consecutive study, 18 eyes were enrolled from 17 pediatric patients (15 boys and 2 girls) with a mean age of 14.44 ± 1.98 years. Manifest refraction, best-corrected visual acuity (BCVA), steepest meridian keratometry (K1), flattest meridian keratometry (K2), maximum keratometry (Kmax), thinnest corneal thickness (TCT), posterior central elevation (PCE), and posterior mean elevation (PME) were measured before and after ATE-CXL. The patients were followed-up at 1, 6, and 12 months. Repeated measures analysis of variance was used for statistical analysis. *P* < 0.05 was considered statistically significant.

**Results:**

There were no complications in any case during or after ATE-CXL. BCVA improved from 0.64 ± 0.32 preoperatively to 0.69 ± 0.32 at 1-year postoperatively. The Kmax value was 56.67 ± 9.60 D before the treatment and 56.19 ± 8.55 D, 56.08 ± 8.85 D, and 55.94 ± 8.46 D at 1, 6, and 12 months postoperatively, respectively. No statistically significant differences were present in K1, K2, Kmax, PCE, and TCT before and after ATE-CXL during the 12-month follow-up (*P* > 0.05).

**Conclusions:**

ATE-CXL is a safe and effective treatment in pediatric progressive keratoconus patients. The long-term effects need further observation.

**Trial registration:**

Retrospectively registered. Registration number: ChiCTR-OIC-16008181. Registered 29 March 2016.

## Background

Keratoconus is a bilateral, noninflammatory, progressive corneal ectasia. The clinical characteristics of keratoconus are progressive thinning and steepening of the cornea, leading to irregular astigmatism and loss of visual acuity [1]. Keratoconus typically presents in the teenage years and progresses until the third or fourth decade [2]. The disease typically is more advanced in younger patients and progresses more rapidly when compared with adults [3, 4]. Therefore, to avoid the need for corneal transplantation, it is imperative to stop the progression of the disease in childhood.

Corneal collagen cross-linking (CXL) can halt the progression of keratoconus by increasing the biomechanical rigidity of the corneal stroma via an interaction of riboflavin and ultraviolet radiation (UV) [5, 6]. Many publications have reported on the safety and efficacy of CXL in the treatment of pediatric keratoconus patients [7–10], but treatment by accelerated transepithelial corneal collagen cross-linking (ATE-CXL) [11, 12], which maintains the integrity of the corneal epithelium layer in pediatric keratoconus patients with a higher irradiation intensity of UV light and a reduced duration of irradiation, has not reported. The present study aimed to assess the safety and efficacy of ATE-CXL in children with progressive keratoconus.

## Methods

### Subjects

All subjects in the present study were recruited from patients who underwent ATE-CXL at Eye and ENT Hospital of Fudan University in Shanghai, China. Criteria for inclusion were age = 10–17 years, progressive keratoconus (≥1.00 D increase in maximal keratometry in the last year, or ≥1.00 D increase in astigmatic degree in the last year), minimal corneal thickness ≥ 400 μm. Exclusion criteria were a history of ocular disease (except keratoconus), previous ocular surgeries, and a history of systemic diseases. Finally, 18 eyes of 17 patients (male:female = 15:2) with a mean age of 14.44 ± 1.98 years were enrolled in this study. The mean spherical error of all the subjects was − 4.17 ± 3.88 D. The mean cylindrical error was − 3.35 ± 2.44 D, and the mean spherical equivalent refraction was − 5.84 ± 4.13 D. Every patient underwent a 1-year follow-up after the operation.

This study adhered to the tenets of the Declaration of Helsinki and was approved by the Ethics Committee of the Eye and ENT Hospital of Fudan University in Shanghai, China. Written informed consent was obtained from at least one parent or legal guardian of each subject after a detailed explanation of the procedure, and all the procedures were carried out with the subjects’ consent.

### Ophthalmologic examinations

All patients underwent slit-lamp biomicroscope examination, best corrected visual acuity (BCVA) and manifest refraction assessment, preoperatively and at 1, 6, and 12 months postoperatively. Steepest meridian keratometry (K1), flattest meridian keratometry (K2), maximum keratometry (Kmax), and thinnest corneal thickness (TCT) were measured by the Pentacam imaging system (Oculus GmbH, Wetzlar, Germany) before the treatment and at each follow-up time point.

Posterior elevation data for the cornea were also obtained by the Pentacam software. The reference best-fit sphere (BFS) was defined in the central 8-mm region of the cornea, which was set to be the same across the images from each patient. Posterior central elevation (PCE) and posterior mean elevation (PME) were measured in the central 4-mm area above the BFS. The change in the posterior elevation (ΔPCE and ΔPME) was found by subtracting preoperative data from postoperative data for each patient.

### Surgical procedures

All surgeries were performed by the same surgeon. Before the surgery, topical anesthetic eye drops were applied. After a lid speculum was used, a trephine (Model 52503B; 66 vision Tech Co, Ltd., Suzhou, China) was placed in the center of the cornea. ParaCel Solution (0.25% riboflavin-5-phosphate, hydroxylpropyl methylcellulose, NaCl, ethylenediaminetetraaceticacid, Tris, and benzalkonium chloride; Medio-Haus-Medizinprodukte GmbH, Kiel, Germany) was dripped into the trephine to cover the corneal epithelium for 4 min. The cornea was then continually infiltrated with Vibex-Xtra Solution (riboflavin phosphate 2.80 mg/mL and NaCl, Avedro, Inc.) for 6 min. After the cornea was rinsed with balanced salt solution (BBS), UV treatment was administered using Avedro’s KXL System (Avedro, Inc) for 5 min and 20 s with 365-nm UV-A light and 45 mW/cm2 irradiation in the pulsed mode (one second on, next second off). BBS was used to keep the ocular surface moist during irradiation. A bandage contact lens was applied after the procedure. Postoperative medications included levofloxacin (4 times daily for 1 week), 0.1% fluorometholone (7 times daily initially, then gradually reduced for 3 weeks), and artificial tears (4 times daily for 4 weeks).

### Statistical analysis

Statistical analysis was performed using the Statistical Package for the Social Sciences (SPSS, Inc., Armonk, NY). Continuous parameters were described as mean ± standard deviation. Repeated measures analysis of variance (ANOVA) with Bonferroni-adjusted post hoc comparisons and Friedman rank test were performed to evaluate the significance of differences between preoperative and postoperative data. A *P* value less than 0.05 was considered statistically significant.

## Results

All procedures were completed successfully, and no intraoperative or postoperative complications were observed. All corneas maintained integrity, but had mild to moderate edema on the first postoperative day. There were no cases of corneal haze or infection.

Refractive parameters at 1, 6, and 12 months of follow-up showed the results displayed in Table 1. At 12-month postoperatively, improvement in the BCVA was noted in 7 (38.89%) eyes, stabilization in 8 (44.44%) eyes, and worsening in 3 (16.67%) eyes. There were no statistically significant differences in SD, CD, SE and BCVA before and after ATE-CXL over the 12-month follow-up (*P* > 0.05).

**Table 1:** Changes in refractive parameters after ATE-CXL(*n* = 18)

	Preoperative	Post 1mo	Post 6mo	Post 12mo
SD	−4.17 ± 3.88	−4.19 ± 3.50	−4.29 ± 3.71	−4.71 ± 4.14
CD	−3.35 ± 2.44	−3.35 ± 2.71	−3.42 ± 2.56	−2.99 ± 2.49
SE	−5.84 ± 4.13	−5.87 ± 3.98	−6.00 ± 4.10	−6.20 ± 4.41
BCVA	0.64 ± 0.32	0.69 ± 0.30	0.64 ± 0.29	0.69 ± 0.32

*SD* spherical degree, *CD* cylindrical degree, *SE* spherical equivalent, *BCVA* best corrected visual acuity

Changes in topographic parameters after ATE-CXL are shown in Table 2. At 12 months postoperatively, the Kmax value decreased in 11 (61.11%) eyes, stabilized in 1 (5.56%) eye, and increased in 6 (33.33%) eyes. There were no statistically significant differences in K1, K2, Kmax, or TCT before and after ATE-CXL over the 12-month follow-up (*P* > 0.05).

**Table 2:** Changes in topographic parameters after ATE-CXL(*n* = 18)

	Preoperative	Post 1mo	Post 6mo	Post 12mo
K1	45.70 ± 3.96	45.79 ± 4.14	45.47 ± 3.65	45.76 ± 3.61
K2	49.22 ± 5.83	49.46 ± 5.81	49.08 ± 5.39	49.14 ± 5.64
Kmax	56.67 ± 9.60	56.19 ± 8.55	56.08 ± 8.85	55.94 ± 8.46
TCT	473.50 ± 36.19	473.33 ± 40.14	473.61 ± 39.28	472.72 ± 34.77

*K1* teepest meridian keratometry, *K2* flattest meridian keratometry, *Kmax* Maximum keratometry, *TCT* thinnest corneal thickness

**Fig. 1:**
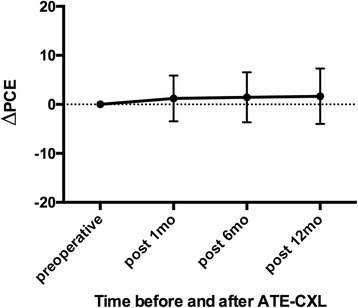
Mean and standard deviation of ΔPCE values at different follow-up times (PCE = posterior central elevation)

**Fig. 2:**
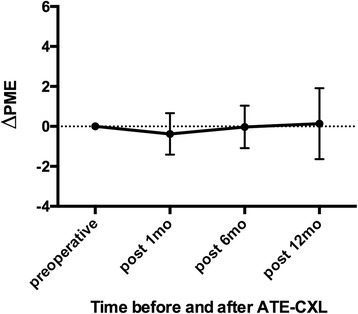
Mean and standard deviation of ΔPME values at different follow-up times (PME = posterior mean elevation)

**Fig. 3:**
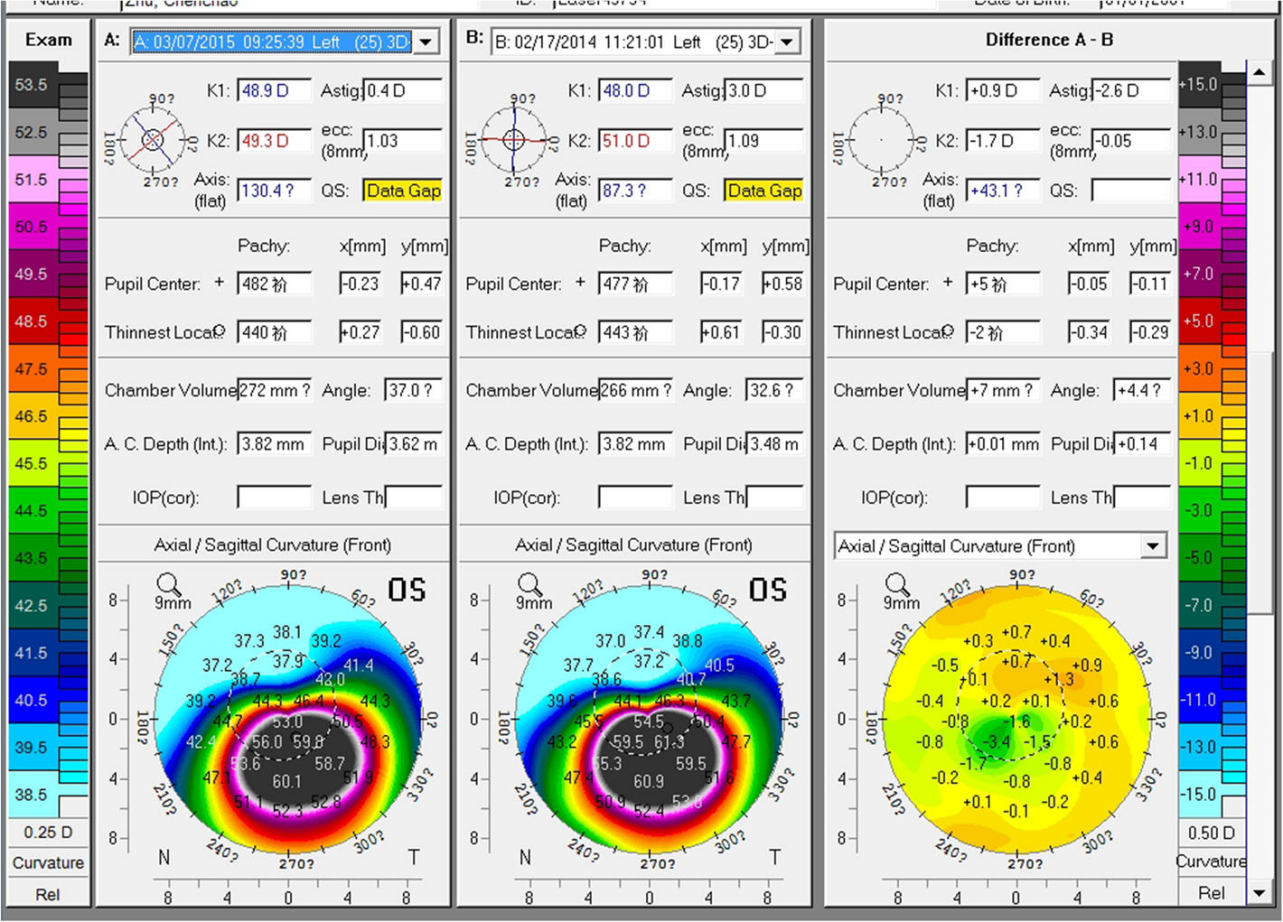
One-year topographic map changes in a typical case. (Comparison of topographic maps preoperatively and at 1 year postoperatively, showing an increase in K1 of 0.9 D, and a decrease in K2 of 1.7 D)

Changes in PCE and PME (ΔPCE and ΔPME; ∆ values were defined by subtracting preoperative data from postoperative data) after ATE-CXL are shown in Figs. 1 and 2. There were no statistically significant differences between ΔPCE and ΔPME over the 12-month follow-up (*P* > 0.05). The topographic map changes in the typical case are shown in Fig. 3.

## Discussion

Although keratoconus is frequently diagnosed during or after adolescence, the ectatic process begins at a much younger age. When it manifests at a pediatric age, progression is more rapid, leading to severe visual impairment with a possible need for corneal transplantation [4, 13]. Corneal CXL is a relatively new and promising treatment that halts the progression of the disease at the corneal ectasia stage. Several studies [7–10] have provided evidence that CXL is safe and effective in slowing or halting the progression of keratoconus,but most of these studies examined conventional CXL (C-CXL). To our knowledge, this study is the first to report the safety and efficacy of ATE-CXL in pediatric keratoconus patients.

All subjects in the present study had progressive keratoconus with an increase in Kmax of 1.00 D or more, or an increase in astigmatism degree greater than 1.00 D. This study showed that the value of BCVA increased from 0.64 ± 0.32 preoperatively to 0.69 ± 0.32 postoperatively, and Kmax decreased from 56.67 ± 9.60 preoperatively to 55.94 ± 8.46 postoperatively at the 1-year follow-up, suggesting that ATE-CXL is an effective procedure that can halt the progression of keratoconus in pediatric patients. In previous studies of C-CXL for the pediatric keratoconus throughout the 1-year follow-up, Stephanie et al. [9] found that BCVA in 39 eyes changed from 0.34 ± 0.27 to 0.34 ± 0.23, and Kmax decreased from 48.49 ± 5.44 D to 48.24 ± 4.47 D, while Ömür et al. [14] found that Kmax in 40 eyes decreased from 58.4 ± 5.5 D to 57.6 ± 6.0 D, which are similar to our results. Previous studies on ATE-CXL for progressive keratoconus have been conducted in adults, and this study is the first to report the effectiveness of ATE-CXL for pediatric keratoconus.

In this study, the values of K1, K2, Kmax and TCT tended to be stable over 1 year of follow-up, suggesting that ATE-CXL showed good stability in the treatment of progressive keratoconus in pediatric patients. Previous studies have reported that after C-CXL, the corneal thickness of patients was significantly decreased compared to the preoperative values, [7, 15] while in our study, we found that ATE-CXL could keep the corneal thickness stable at each follow-up time point because of its retention of the corneal epithelium. The previous studies typically evaluated the stability of CXL for keratoconus in terms of visual acuity, corneal thickness and keratometry value, and the corneal posterior elevation could also be used as a reliable way of evaluating the stability of the corneal structure. A positive change of the posterior corneal surface indicated an ectatic change of the cornea. This study showed that 1 year postoperatively, the PCE and PME values had no statistically significant differences from the preoperative values, which indicates that ATE-CXL could stabilize the structure of the cornea by preventing corneal expansion. To our knowledge, this is the first prospective clinical study in which corneal posterior elevation had been observed in keratoconus after ATE-CXL.

The current study reported no intraoperative and postoperative complications in pediatric patients, indicating the safety of ATE-CXL. Corneal epithelial defects and corneal haze have been reported for C-CXL treatment of keratoconus [16, 17], but these were not observed for the ATE-CXL treatment. C-CXL treatment with low UV radiation energy (3 mW/cm2) and long exposure time (30 min) required a long recovery time for the patients, because of removal of the epithelium and the resulting risk of haze. However, ATE-CXL in our study not only maintained the integrity of the corneal epithelium, but also shortened the time of infiltration (10 min) and irradiation (5 min and 20 s). Therefore, ATE-CXL produced mild corneal irritation symptoms and light postoperative reactions, so that patients felt more comfortable in the treatment process, which is more suitable for patients with thin corneas and pediatric patients.

This study demonstrated that ATE-CXL is a safe and effective treatment for progressive keratoconus in pediatric patients. The principle limitation of this study was that the sample size was small, and a longer follow-up of ATE-CXL for pediatric patients is needed to validate the findings.

## Conclusions

ATE-CXL is a safe and effective treatment for pediatric progressive keratoconus patients. The long-term effects of ATE-CXL in pediatric patients need further observation.

## Abbreviations

ATE-CXL: Accelerated transepithelial corneal collagen cross-linking
BCVA: Best corrected visual acuity
C-CXL: Conventional corneal collagen cross-linking
CD: Cylindrical degree
K1: Steepest meridian keratometry
K2: Flattest meridian keratometry
Kmax: Maximum keratometry
PCE: Posterior central elevation
PME: Posterior mean elevation
SD: Spherical degree
SE: Spherical equivalent
TCT: Thinnest corneal thickness
